# Persistently Elevated C-Reactive Protein Levels and Low Body Mass Index Are Associated with a Lack of Improvement in Bone Mineral Density in Crohn’s Disease

**DOI:** 10.3390/nu16172827

**Published:** 2024-08-23

**Authors:** Eduard Koifman, Meytal Krasnopolsky, Itai Ghersin, Matti Waterman

**Affiliations:** 1 Inflammatory Bowel Diseases Service, Gastroenterology Institute, Rambam Health Care Campus, Haifa 3109601, Israel; meytalmakh@gmail.com (M.K.); ighersin@gmail.com (I.G.); m_waterman@rmc.gov.il (M.W.); 2 The Ruth and Bruce Rappaport Faculty of Medicine, Technion—Israel Institute of Technology, Haifa 3109601, Israel

**Keywords:** Crohn’s disease, osteoporosis, bone mass density (BMD), body mass index (BMI), C-reactive protein (CRP)

## Abstract

Background: Osteoporosis prevalence is increased in Crohn’s disease (CD). Its pathogenesis in these patients is incompletely understood. Objectives: To identify factors associated with decreased bone mineral density (BMD) status in CD patients on a time-line course. Methods: A retrospective study was performed that followed CD patients who underwent at least two bone mineral density scans (DEXAs). Follow-up began one year prior to the first DEXA test and lasted at least one year after a second test. Possible correlations between baseline and follow-up variables and changes in BMD status were examined. Change in BMD was defined as a transition from one bone density category to another (normal vs. osteopenia vs. osteoporosis). Binary variables were assessed using the Cochrane–Armitage test. Categorical variables were assessed using the chi-squared test. A multivariate analysis was performed. Results: The study included 141 patients. At baseline, 33 patients (23.4%) had normal BMD, 75 (53.2%) had osteopenia, and 33 (23.4%) had osteoporosis. Patients with low BMD had a lower baseline BMI compared to those with normal BMD (*p* < 0.0001). After a median follow-up of 48 months (IQR 29–71), BMD status worsened in 19 (13.5%) patients, whereas in 95 (67.3%) and 27 (19.1%) patients, BMD remained unchanged or improved, respectively. On the multivariate analysis, elevated median CRP throughout follow-up (OR = 0.8, 95% CI: 0.68–0.93) and low baseline BMI (OR = 0.9, 95% CI: 0.83–0.98) were associated with a lack of BMD status improvement. Conclusions: Persistently elevated CRP and low BMI are associated with a lack of improvement in BMD. These findings underscore the importance of effective inflammation control and nutritional support to maintain and improve bone health.

## 1. Introduction

Crohn’s disease (CD) is a chronic condition characterized by inflammation of the digestive tract. It affects millions of individuals worldwide, with increasing prevalence around the world among all age groups [1].

Patients with Crohn’s disease experience a reduced quality of life and potential disability not only due to a persistent gut inflammation but also due to extraintestinal manifestations of the disease including reduced bone health. Osteoporosis and osteopenia are defined as a reduced bone mineral density (BMD), assessed by a dual-energy X-ray absorption (DEXA) scan. In adults, a T-score lower than −2.5 SD determines osteoporosis while a T-score between −1 SD and −2.5 SD determines osteopenia [2]. 

Osteoporosis is often an under-recognized complication, although it is associated with a two- and three-fold increased risk of spinal and hip fractures, respectively, compared to the general population [3]. Several longitudinal studies have found a T-score of <−2.5 in 5–37% of IBD patients [4].

The pathogenesis of reduced BMD in Crohn’s disease is probably multifactorial and may be attributed to an interplay between several factors such as continuous systemic inflammation, frequent corticosteroid (CS) use, low body mass index (BMI), smoking, malabsorption of vitamins D and K and calcium, malnutrition, reduced physical activity, and genetic factors [5].

While research has established a clear association between Crohn’s disease and an increased risk of osteoporosis, data on longitudinal bone density changes in CD patients are limited. The management of IBD has undergone a significant transformation in recent years with the advent of biological therapies. These targeted medications have revolutionized treatment, offering improved clinical outcomes and quality of life for many patients. However, despite their remarkable efficacy in addressing IBD symptoms, the impact of biological therapies on bone health remains largely unexplored. This scarcity of information poses a significant challenge for clinicians seeking to optimize bone health management in IBD patients.

This study aimed to better understand the risk factors associated with decreased bone mineral density in Crohn’s disease and track changes in bone density status in the era of modern IBD care.

## 2. Materials and Methods

We conducted a descriptive observational retrospective study based on the database at our center. 

Computerized records and the manual logs of patients treated at our clinic were reviewed, identified using the MDCLONE software (https://www.mdclone.com/, accessed on 28 July 2024). The search was conducted using the keywords: “bone density”, “osteoporosis”, “osteopenia”, “T SCORE”. 

Patients were eligible for enrollment if they had a confirmed diagnosis of Crohn’s disease, were followed up at our institution between January 2000 and February 2024, had at least two bone density tests during this period, and were over 18 years old at the time of the first examination.

The primary outcome was a change in bone mineral density (BMD) status at the end of the study period. We defined this change as “improvement”, “deterioration”, or “no change”, when a transition was observed from normal BMD status to osteopenia or from osteopenia to osteoporosis or vice versa, based on results of two consecutive DEXA scans. Bone status was classified as normal, osteopenia, or osteoporosis according to the accepted definitions in the literature [2] as mentioned above. The follow-up period was set to begin one year before the first DEXA test and to end one year after the last one.

In addition to the primary outcome, data on additional variables were collected and categorized into baseline variables and follow-up variables. As fractures were not reliably recorded in the patients’ records and only few events were reported, they were not included in this analysis.

Baseline variables included demographic variables such as gender, age, initial BMI (calculated at each time point as weight (kg)/height^2^ (meters)), and disease-related variables such as age at diagnosis, disease duration at the beginning of follow-up, disease behavior, and laboratory markers at the beginning of the follow-up period including vitamin D, calprotectin, and CRP. 

C-reactive protein (CRP) was measured relative to the upper limit of normal (ULN). Vitamin D levels were defined as serum vitamin D25-OH level, with deficiency and insufficiency determined as <20 ng/mL and <30 ng/mL, accordingly, as defined by the Endocrine Society’s 2011 consensus guidelines [6].

Follow-up variables included clinical variables such as hospitalizations, surgical procedures, changes in BMI (calculated as BMI measured at latest time point during follow-up minus BMI at baseline), smoking status, and laboratory markers such as median CRP, mean vitamin D and fecal calprotectin, immunosuppressive medication exposure, and vitamin D supplementation.

Immunosuppressive medication exposure was deemed positive if continued for at least 3 months. Exposure to corticosteroids (CSs) was classified into three groups: Patients in the “no CS treatment” group were not exposed to any CS during the study period, those in the “limited dose treatment” received either doses below 20 mg of prednisone or above 20 mg of prednisone but for less than 3 months or budesonide for more than 3 months. The third group included patients with “prolonged CS use” defined as patients treated with prednisone at doses above 20 mg for more than 3 months.

This study was performed in accordance with institutional Helsinki approval number RMB-D-0411-21.

### Statistical Methods

First, the correlation between baseline data and initial bone density status was examined. The association between continuous variables and BMD status was tested using the Kruskal–Wallis test. For variables with a symmetrical distribution, a regression analysis was conducted with bone density status as an ordinal variable to generate a *p*-value for the trend. Variables with missing values in more than 20% of patients were excluded from the statistical analysis.

The relationship between bone density status and binary variables was tested using the Cochrane–Armitage test, and categorical variables were examined using the chi-squared test.

Correlations between baseline variables and changes in bone density status were analyzed, with patients divided into three groups: the “improvement” group, the “deterioration” group, and the “no change” group, using similar tests as in the first analysis.

Correlations between follow-up variables and changes in bone density status (ordinal response variable) were examined using a proportional odds ordinal model with backwards selection (with criteria *p* < 0.2). Results are presented for the model with the selected variables, anti-TNF and corticosteroid treatment.

## 3. Results

**Figure 1:**
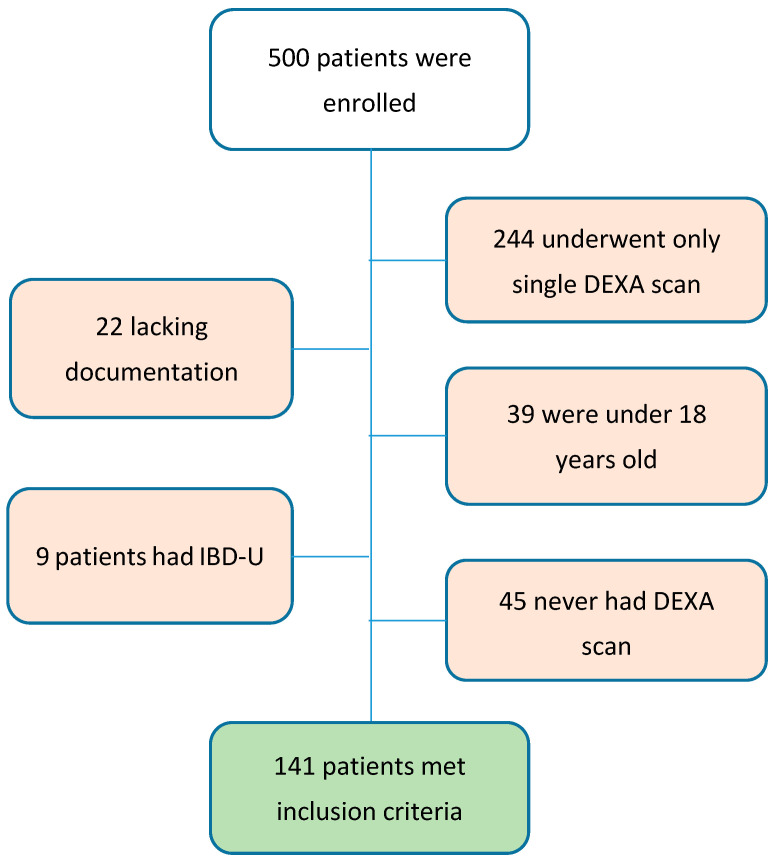
Patient enrollment flow.

A total of 500 medical records were reviewed for the study, 141 patients met the inclusion criteria, and 70 (49.6%) of them were females (Figure 1). At follow-up start (Table 1), the median age was 34 years (IQR 24–47), with median disease duration prior to inclusion of 6 years (IQR 1–14). Median CRP at baseline was 1.4 ULN, and median vitamin D level was 54.1 nmol/L. At baseline, the median (IQR) body mass index (BMI) was 22.4 (19.2–26.2) kg/m^2^, with 28 (19.9%) patients categorized as underweight (BMI > 18.5 kg/m^2^), 68 (48.2%) patients were normal (BMI = 18.5–24.9 kg/m^2^), 34 (24.1%) patients were overweight (BMI = 25–29.9 kg/m^2^), and 10 (7.1%) patients were obese (BMI ≥ 30 kg/m^2^). 

At baseline, 33 patients (23.4%) had normal BMD, 75 (53.2%) had osteopenia, and 33 (23.4%) had osteoporosis. A significant difference in BMI was observed between these groups, and patients with normal bone density had a higher BMI compared to patients with osteopenia (*p* < 0.01) and osteoporosis (*p* < 0.0001), while patients with osteopenia had a higher BMI than the osteoporosis group (*p* < 0.001). No other statistically significant correlations between baseline characteristics and baseline BMD status were found (Table 2). Extraintestinal manifestations (EIMs) were present in 40 (28.4%) patients in our cohort. We looked for musculoskeletal and liver EIMs, as they could potentially alter BMD status. Only 1 patient in our cohort had primary sclerosing cholangitis, while 9 patients had peripheral arthritis, 7 had axial arthropathy, and 25 had arthralgia. No correlation was found between the presence of these EIMs and BMD status (Table 2).

The follow-up period continued for a median of 48 (IQR 29–71) months. The bone density status worsened in 19 patients (13.5%), remained unchanged in 95 (67.3%), and improved in 27 patients (19.1%). During the follow-up, 81 (57.4%) patients were hospitalized at least once, 41 (29.1%) underwent IBD-related surgery, 75 (53.2%) were exposed to anti-TNF agents, 74 (52.5%) to thiopurines, 24 (17.0%) to vedolizumab, and 18 (12.8%) to ustekinumab. Low-dose exposure to corticosteroids was documented in 34 (24.1%) patients, while 48 (34%) were given high-dose steroids. 

Elevated median CRP throughout follow-up (OR 0.8, 95% CI: 0.68–0.94, *p* < 0.01) and low initial BMI (OR 0.9, 95% CI: 0.83–0.98, *p* < 0.01) were associated with a decreased likelihood of bone density improvement. Oppositely, weight gain was modestly associated with an improvement in bone density (OR 1.1, 95% CI: 098–1.23, *p* = 0.09) (Table 3).

On the multivariable analysis of the association of baseline and follow-up variables and change in BMD status (Table 4), only baseline BMI and higher median CRP levels during F/U were independently associated with a change in BMD.

## 4. Discussion

Osteoporosis and osteopenia are quite common in Crohn’s disease patients, including in our cohort. Furthermore, in our follow-up, about one-sixth of patients experienced a decline in bone density over the course of the study. Persistently elevated CRP levels were associated with a lack of improvement in bone density.

The mechanisms that predispose patients with Crohn’s disease (CD) to osteoporosis are probably multifactorial. These factors include, among others, vitamin D deficiency, systemic inflammation, malnutrition, use of oral CSs, and decreased physical activity. Although it is challenging to determine the relative contribution of each factor, the final result is a high prevalence of osteoporosis in this population, ranging from 7% to 15% in different series [7,8]. The prevalence of osteoporosis in our study was even higher, as 23% of patients suffered from osteoporosis, and an additional 52% had osteopenia at the beginning of the follow-up period. It is possible that such a high prevalence of reduced bone health results because of a long time-lag (median 6 years) from initial diagnosis to the follow-up start. Another possible explanation is a selection bias of patients with at least two DEXA scans, which reflects physicians’ decisions to monitor bone density in either patients with reduced bone density or relatively severe long-standing CD. This bias may be reflected in the observed high admission and surgery rates, with 57% of patients being admitted at least once and about 34% operated on during the follow-up period. According to a study by Tsai et al., hospitalization was among the highest risk factors associated with osteoporosis in Asian patients with IBD [9]. 

Elevated serum CRP over the study period that may be a reflection of active gut inflammation was associated with a lack of improvement in BMD (OR 0.8, CI: 0.68–0.93). To the best of our knowledge, this is the first study to show that persistently elevated CRP is associated with time-dependent bone density deterioration in the Crohn’s disease population. This association indicates that active disease is likely a contributing factor to bone loss in patients with Crohn’s disease. Moreover, studies have shown an association of CRP with the pathogenesis of osteoporosis in the general population, and the levels of inflammatory factors such as hs-CRP were detected to be increased in osteoporosis patients [10]. 

A significant correlation was found between baseline BMI and baseline BMD, and patients with low bone density status had a significantly lower initial BMI compared to those with normal bone density status (median 26 vs. 18.5 for osteoporosis and 22.4 for osteopenia). This aligns with the known association between low BMI, malnutrition, and secondary osteoporosis [11]. BMI reflects nutritional status, and low BMI may also suggest poorly controlled disease. Additionally, BMI has been found to be a crucial factor in determining BMD in both IBD patients and the general population. A longitudinal study of IBD patients demonstrated a significant negative correlation between BMI and the rate of bone loss [12]. 

Though TNFα is also linked to the pathogenesis of osteoporosis, we did not find a beneficial effect of anti-TNF agents on bone density status. A 2011 literature review showed improved bone density in Crohn’s patients treated with infliximab (monoclonal anti-TNF-α antibody), but these studies had small sample sizes and short follow-up periods [13]. Conversely, a recent Polish study found no association between TNF-α inhibitor treatment and osteoporosis in IBD patients [7]. The lack of robust longitudinal data on BMD changes in IBD patients treated with biological therapies stems from several factors. Firstly, the relatively recent introduction of these medications has limited the duration of follow-up studies, hindering the assessment of long-term effects on bone health. Secondly, the heterogeneity of IBD and the variability in individual responses to treatment further complicate the analysis of bone density trajectories. Long-term, well-controlled trials are necessary to comprehensively assess the effects of TNF blockade on bone mineral density.

We did not find a significant relationship between corticosteroid treatment and BMD changes, though there was a clear trend indicating a negative correlation between corticosteroid dose and BMD. It is difficult to distinguish disease activity from CS use in terms of causal impact on BMD because they are so closely interrelated. The link between CSs and bone loss is well established. However, longitudinal studies on bone loss rates in IBD show conflicting results regarding the impact of CSs [7,12,14].

Active smoking was associated with a decrease in bone density on the univariate analysis (Table 1). Smoking is a well-known risk factor for reduced bone density in the general population, possibly affecting vitamin D and calcium levels [15]. In Crohn’s patients, smoking is linked to a more severe disease course, leading to significant absorption issues, aggressive treatment, and prolonged immobility, all contributing to bone status deterioration [16]. Smoking was also associated with bone density status deterioration, although this was not statistically significant, possibly due to low sample size (Table 4).

Baseline and median vitamin D levels throughout the follow-up were not associated with changes in bone density. Interestingly, contrary to common belief, on a univariate analysis, an increase in vitamin D was associated with a deterioration rather than an improvement in BMD. We speculate that patients with more active and severe disease were more likely to take vitamin D supplements. The fact that the relationship ceased to be significant in a multi-variable model provides support for this assumption. Furthermore, Leslie et al. reported that just 21.8% of IBD patients achieved optimal vitamin D levels (>30 ng/mL), underscoring the necessity to adjust dosages for those currently receiving treatment and to initiate treatment for patients with risk factors [17].

Our study has some important limitations. As it is a retrospective study, it relies on recorded patient data, which may be incomplete or inaccurate. This has led to the exclusion of fractures as an endpoint in this study, despite being the most clinically relevant outcome of osteoporosis. The small sample sizes, especially in the bone density status change groups, may impact results. Treating physicians were not blinded to patients’ DEXA scans and disease severity, and therefore their decisions may have impacted bone health during follow-up. Moreover, non-documented, non-uniform variations in the implementation of professional societies’ recommendations on IBD patients with reduced bone density may have increased, confounding with other variables. Additionally, the requirement for two bone density tests may have biased the selection towards patients with a more severe disease course or increased risk for BMD deterioration, indicating the need for repeated testing. 

## 5. Conclusions 

In conclusion, low BMI and active smoking were identified as risk factors for impaired bone density status. A trend, though not statistically significant, was observed between BMI improvement and bone density status improvement. Persistently elevated CRP, potentially a marker of ongoing inflammation, was linked to bone density deterioration. While the associations of low BMI and persistent CRP with osteoporosis have been reported in the general population [10], studies looking into the impact of persistent inflammation and the pathogenesis of osteoporosis in IBD are required. Moreover, prospective data showing that good control of inflammation and nutritional status in IBD will lead to prevention of osteoporosis and fractures are needed. Current recommendations for bone density screening in Crohn’s patients are based on general population parameters. The factors highlighted in this study could be relevant to both Crohn’s patients and the general population. It should be noted that in our cohort, despite physician awareness of the implications of decreased bone density, about one-sixth of patients experienced bone density decline, underscoring the importance of preventive interventions such as smoking cessation, effective inflammation control, avoidance of steroids, and improving nutritional support, especially for patients with low BMI.

Nevertheless, further comparative research is needed to corroborate our findings.

High-quality, long-term studies are essential to enhance our understanding of the interplay between IBD, biological therapies, and bone health. This understanding will enable clinicians to make informed treatment choices, improve bone health management, and ultimately enhance the well-being of patients with IBD.

## Figures and Tables

**Table 1:** Baseline characteristics of the patients included in this study.

	Min	Max	IQR	Median
Age at baseline [years]	18	73	24–47	34
Age at diagnosis [years]	9	68	17–36	23
Disease duration [years]	0	35	1–14	6
BMI [kg/m^2^]	12.9	39.3	19.2–26.2	22.4
CRP [value/ULN]	0.1	36.3	0.5–4.3	1.3
FCP [µg/g stool]	5	7810	45–300	150
Vitamin D [nmol/L]	16.5	157	39.0–69.6	54.1
			n (%)	
Gender				
Female			70 (49.6)	
Male			71 (50.4)	
Smoking status				
Yes 32 (22.5)				
No 110 (77.5)				
Disease location * (Montréal criteria)				
Ileal (L1) 60 (42.3)				
Colonic (L2) 21 (14.8)				
Ileocolonic (L3) 61 (43.0)				
Upper GI (L4) 10 (7.0)				
Perianal (P) 48 (33.8)				
Disease behavior *				
Inflammatory (B1)			67 (47.2)	
Fibrostenotic (B2)			39 (27.5)	
Penetrating (B3)			36 (25.3)	
Extraintestinal manifestations				
Peripheral arthritis			9 (6.3)	
Axial arthropathy			7 (4.9)	
Arthralgia			25 (17.6)	
Primary sclerosing cholangitis			1 (0.7)	
Perianal involvement			48 (33.8)	

IQR—25–75% interquartile range BMI—body mass index, CRP—C-reactive protein, FCP—fecal calprotectin, ULN—upper limit of normal, *—at the beginning of follow-up.

**Table 2:** Associations between baseline characteristics and baseline BMD status.

	Normal (n = 33)	Osteopenia (n = 75)	Osteoporosis (n = 33)	*p*-Value
Age at diagnosis [years] Median (IQR)	23 (17–37)	24.5 (17.7–35)	21 (16–31)	0.40 ^a^
Age at baseline [years] Median (IQR)	33 (22–47)	33 (27.5–48)	27(22–46)	0.52 ^a^
IBD duration [years] Median (IQR)	7 (2–14)	5 (0–14)	7 (3–13)	0.54 ^a^
BMI at baseline [kg/m^2^] Median (IQR)	26 (22.8–28.7)	22.4 (20–26)	18.4 (17.3–21)	<0.01 ^a^
CRP at baseline [value/ULN] Median (IQR)	1.2 (0.4–2.8)	2.2 (0.7–4.8)	1 (0.5–4.1)	0.24 ^a^
Vitamin D at baseline [nmol/L] Median (IQR)	49.3 (40.8–55)	57.4 (38.2–67.5)	60.0 (39.5–76.7)	0.19 ^b^
Gender				
Female, n (%)	14 (45.2)	32 (50.8)	14 (51.9)	0.33 ^b^
Smoking status				
Yes, n (%)	9 (27.3)	18 (23.7)	5 (15.2)	0.24 ^b^
Disease behavior				
Inflammatory (B1), n (%)	15 (45.5)	38 (50.0)	14 (42.4)	0.72 ^a^
Fibrostenotic (B2), n (%)	8 (24.2)	19 (25.0)	12 (36.6)	
Penetrating (B3), n (%)	10 (30.3)	19 (25.0)	7 (21.2)	
Peripheral arthritis, n (%)	2 (6.1)	5 (6.7)	2 (6.1)	0.80
Axial arthropathy, n (%)	1 (3)	5 (6.7)	1 (3)	0.61
Arthralgia, n (%)	6 (18.2)	17 (22.7)	2 (6.1)	0.11

^a^ *p*-value for association, ^b^ *p*-value for trend, IBD = inflammatory bowel disease, BMI = body mass index, CRP = C-reactive protein.

**Table 3:** Univariate analysis of variables during follow-up and the change in BMD status.

	Worsened n = 19	No Change n = 95	Improved n = 27	*p*-Value
Median CRP, median during F/U (IQR) [value/ULN]	1.5 (0.5–3.2)	1 (0.6–2.4)	1 (0.4–1.5)	0.18
Mean calprotectin during F/U, median (IQR) [µg/g]	243 (117–414)	176 (70–428)	150 (37–255)	0.50
ΔVitamin D, mean (IQR) [nmol/L]	10.9 (4.6–15.8)	2.9 (−4.2, 15.1)	−0.4 (−19.5, 4.9)	0.01
ΔBMI, median (IQR) [kg/m^2^]	0.6 (−1.1, 3.3)	1.5 (−0.1, 3.1)	3.4 (1, 4.3)	0.10
Smoking, n (%)	7 (36.8)	23 (24.2)	2 (7.4)	0.02
Hospitalization, n (%)	13 (68.4)	48 (61.5)	20 (83.3)	0.23
Surgery, n (%)	6 (31.6)	25 (32.1)	10 (41.7)	0.54
Vitamin D supplement, n (%)	15 (78.9)	66 (69.5)	22 (81.5)	0.38
Anti-TNF treatment, n (%)	9 (47.4)	49 (51.5)	17 (66.7)	0.29
Thiopurines treatment, n (%)	13 (68.4)	41 (43.2)	20 (74.1)	0.77
Vedolizumab treatment, n (%)	4 (21.1)	19 (20.0)	1 (3.7)	0.10
Ustekinumab treatment, n (%)	2 (10.5)	14 (14.7)	2 (7.4)	0.70
Steroid treatment *				0.24
None, n (%)	3 (15.7)	45 (47.3)	11 (40.7)	
Limited dose, n (%)	6 (31.5)	23 (24.2)	5 (18.5)	
Prolonged use, n (%)	10 (52.6)	27 (28.4)	11 (40.7)	

F/U—follow-up, ΔVitamin D = (follow-up Vitamin D) − (baseline Vitamin D), ΔBMI = (BMI at the end of follow-up) − (BMI at baseline), CRP = C-reactive protein, BMI = body mass index, ULN = upper limit of normal, * “Limited dose treatment”—either doses below 20 mg prednisone or above 20 mg prednisone but for less than 3 months or budesonide for more than 3 months. “Prolonged use”—prednisone at doses above 20 mg for more than 3 months.

**Table 4:** Multivariate analysis of different variables associated with a change in bone mineral density *.

	OR	95% CI	*p* Value
Median CRP during F/U	0.80	**[0.68–0.94]**	**0.0084**
Baseline BMI	0.90	**[0.83–0.98]**	**0.0123**
ΔBMI	1.10	[0.98–1.23]	0.0906
Smoking	0.50	[0.20–1.29]	0.1522
Mean vitamin D	1.02	[0.99–1.04]	0.1195
Corticosteroids exposure	0.97	[0.62–2.95]	0.9044
Anti-TNF treatment	1.35	[0.61–1.54]	0.4498

*—variables with *p* < 0.2 on univariate analysis were included. OR = odds ratio, CI = confidence interval, BMI = body mass index, CRP = C-reactive protein. ΔBMI—change in BMI, variables reaching statistical significance appear in bold.

## Data Availability

The information is not publicly available as it is stored in coded form in the hospital’s databases.
